# Identification of Earthquake Induced Damage Areas Using Fourier Transform and SPOT HRVIR Pan Images

**DOI:** 10.3390/s90301471

**Published:** 2009-03-03

**Authors:** Elif Sertel

**Affiliations:** Istanbul Technical University, Faculty of Civil Engineering, Department of Geodesy and Photogrammetry, Maslak, 34469, Istanbul, Turkey

**Keywords:** Remote sensing, earthquake, Fourier, damage assessment

## Abstract

A devastating earthquake with a magnitude of Mw 7.4 occurred on the North Anatolian Fault Zone (NAFZ) of Turkey on August 17, 1999 at 00:01:39 UTC (3:01 a.m. local time). The aim of this study is to propose a new approach to automatically identify earthquake induced damage areas which can provide valuable information to support emergency response and recovery assessment procedures. This research was conducted in the Adapazari inner city, covering a 3 × 3 km area, where 11,373 buildings collapsed as a result of the earthquake. SPOT high resolution visible infrared (HRVIR) Pan images obtained before (25 June 1999) and after (4 October 1999) the earthquake were used in the study. Five steps were employed to conduct the research and these are: (i) geometric and radiometric correction of satellite images, (ii) Fast Fourier Transform (FFT) of pre- and post-earthquake images and filtering the images in frequency domain, (iii) generating difference image using Inverse Fast Fourier Transform (IFFT) pre- and post- earthquake images, (iv) application of level slicing to difference image to identify the earthquake-induced damages, (v) accuracy assessment of the method using ground truth obtained from a 1/5,000 scale damage map. The total accuracy obtained in the research is 80.19 %, illustrating that the proposed method can be successfully used to automatically identify earthquake-induced damage areas.

## 1. Introduction

Turkey is one of the most seismically active regions on the earth. Different fault systems in Anatolia and the surrounding regions were created due to the complex plate interactions among Arabia, Eurasia and Africa [1]. The North Anatolian Fault Zone (NAFZ) and the East Anatolian Fault Zone (EAFS) are the main strike-slip fault belts in Turkey where several earthquakes have occurred, resulting in huge numbers of fatalities over the past several hundred years (Table 1).

Earthquakes and other natural hazards can cause disasters of uncontrollable magnitude when they hit large urban areas. Emergency response and early recovery assessment in earthquakes require rapid and reliable damage assessment and loss estimation. In the case of suddenly occuring earthquakes, remote sensing data can be reliably used to create fast draft damage maps of the affected urban areas which provide valuable information to support emergency response teams and decision making during the recovery process. Remotely sensed images ranging from very high resolution to medium resolution have been widely used to derive information and estimation for damage assessment [2–7]. Morevover, multitemproral remote sensing data can serve as a basic data set to support post-disaster planning.

Different remote sensing methods have been used by many scientists to identify earthquake-induced damage areas. Sertel *et al.* [5] investigated the relationship between semivariogram metrics and degree of earthquake damage using transects over an earthquake area. Turker and San [7] used differences between merged pre- and post-event Satellite Pour l’Observation de la Terre (SPOT) high resolution visible (HRV) data to reveal the location of earthquake-induced changes. Stramondo *et al.* [6] used coherence and correlation maps from Advanced SAR (ASAR) and change maps from advanced space-borne thermal emission and reflection radiometer (ASTER) to analyze the capabilities and limitations of satellite remote sensing to detect damage due to earthquakes. Kaya *et al*. [3] used government statistics and SPOT HRV data to estimate the proportion of collapsed buildings in an earthquake area. Although there have been a lot of earthquake damage assessment studies using different remote sensing methods, there has not been that much research on the application of Fourier Transform to satellite image for an earthquake case. This research focuses on integrated usage of Fourier Transform and level slicing to identify earthquake induced damage areas, also detailed accuracy assessment of proposed method was conducted using 1/5,000 scale damage map data and error matrix analyses.

Fourier transforms have been applied to different remote sensing applications. Lillo-Saavedra *et al.* [8] used Fourier transforms to fuse panchromatic and multispectral data obtained from Landsat ETM+ sensor. Westra *et al.* [9] used Fourier analysis of Moderate Resolution Image Spectrometer (MODIS) time series data to monitor the flooding extent. Pal *et al.* [4] used fast Fourier transform (FFT) filter to extract linear and anomalous patterns. Their results showed that numerous lineaments and drainage patterns could be identified and demarcated by FFT filters.

In this study, the following steps were conducted to accurately identify the location and magnitude of earthquake induced damages in an urban area and to quantify the accuracy of the proposed method: (i) pre- and post-earthquake images of the region were geometrically and atmospherically corrected, (ii) Fast Fourier Transform (FFT) was applied to pre- and post-earthquake images and images were filtered in the frequency domain, (iii) a difference image was generated using Inverse Fast Fourier Transform (IFFT)-pre- and post-earthquake data, (iv) level slicing method was applied to difference image to identify the earthquake-induced damages, (v) accuracy assessment was performed by comparing the results of the proposed method with the 1/5,000 scale damage map of the earthquake area.

## 2. The Study Area and Data

A devastating earthquake with a magnitude of Mw 7.4 occurred on the North Anatolian Fault Zone (NAFZ) of Turkey on August 17, 1999 at 00:01:39 UTC (3:01 a.m. local time). The center of the earthquake was at 40.74 N., 29.86 E. The earthquake struck Kocaeli and surrounding cities, namely Adapazari, Golcuk and Yalova, and brought about massive destruction to these cities and their surrounding rural areas. This was one of the most destructive earthquakes of the Twentieth Century considering the amount of damage and number of casualties. At least 17,118 people were killed, approximately 50,000 injured and the estimated financial loss in Istanbul, Kocaeli and Adapazari was 3 to 6.5 billion U.S. dollars [3, 5, 10].

This research investigates the earthquake induced changes in the city center of Adapazari where the recorded number of collapsed buildings was 11,373 [5]. The district of Adapazari is located in the northeastern part of the Marmara Region, Turkey, covering 29°57′–30°53′ N and 40° 17′–41°13′ E. The population of the Adapazari inner city was 169,099, 184,013, and 172,000 in 1990, 1997, and 2000, respectively [11].

SPOT HRVIR panchromatic images obtained before (25 June 1999) and after (4 October 1999) the earthquake were used in the research. These images have 10 m spatial and 8 bit radiometric resolution. The 1/5,000 scale digital damage map illustrating the degree of earthquake damage was used to analyze the accuracy of the proposed method. This map was produced by local and federal authorities by conducting a field survey on the building base after the earthquake.

## 3. Methodology

### 3.1. Radiometric Normalization and Geometric Correction

Both images were first geometrically corrected into Universal Transverse Mercator (UTM) projection using first order polynomials and appropriate Ground Control Points (GCP) collected from topographic maps. Then, radiometric normalization was employed using a histogram matching algorithm.

### 3.2. Fourier Transform

Any one-dimensional function, *f(x)* (which might be a row or column of pixels), can be represented by a Fourier series composed of some sine and cosine terms and their associated coefficients combination. Different spatial frequencies over an image can be represented by many sine and cosine terms and with their associated coefficients. Fourier series are effective to identify and quantify spatial frequencies [12,13]. Since an earthquake changes the spatial structure of a related area because of collapsed or damaged buildings, roads etc., Fourier series can be used to identify different spatial frequencies in images obtained before and after the earthquake which indeed lead information about the earthquake-induced damages.

The Fast Fourier Transform (FFT) calculation used in this research is shown in the equation 1 [12]:

(1) $$F(u,v)\leftarrow \sum_{x=0}^{M-1}\sum_{y=0}^{N-1}\left[f(x,y)e^{-\frac{j2\pi \mathrm{ux}}{M}-\frac{j2\pi \mathrm{vy}}{N}}\right]$$

where:

*M* = the number of pixels horizontally
*N* = the number of pixels vertically
*u,v* = spatial frequency variables
*e* = 2.71828, the natural logarithm base
*j* = the imaginary component of a complex number

Once the FFT is applied, a raster image from the spatial domain is converted into a frequency domain image. The Fourier image can be edited (mainly using filters) to reduce noise, to identify specific features or to remove periodic features. After editing the Fourier image, it is transformed back into spatial domain using Inverse Fast Fourier Transform (IFFT) equation (Equation 2) [12]:

(2) $$\begin{array}{c} f(x,y)\leftarrow \frac{1}{N_{1}N_{2}}\sum_{u=0}^{M-1}\sum_{v=0}^{N-1}\left[F(u,v)e^{\frac{j2\pi \mathrm{ux}}{M}+\frac{j2\pi \mathrm{vy}}{N}}\right]\\ 0\leq x\leq M-1,0\leq y\leq N-1 \end{array}$$

### 3.3. Difference Image and Level Slicing

A difference image was calculated by subtracting the inverse Fourier transformed post- and pre-earthquake images. The difference image then divided into slices based on the number of bins (10 for this research) using the following equations:

(3) $$x=\frac{{\mathrm{DN}}_{\max}-{\mathrm{DN}}_{\min}}{\mathrm{number}\;\mathrm{of}\;\mathrm{bins}}$$

(4) $${\mathrm{DN}}_{\mathrm{out}}=\frac{{\mathrm{DN}}_{\mathrm{in}}-{\mathrm{DN}}_{\min}}{x}$$

where:

DN_max_ = Maximum value of Digital Numbers
DN_min_ = Minimum value of Digital Numbers
DN_in_ = Input Digital Number
DN_out_ = Output Digital Number after level slicing
DN_out_ values obtained after the level slicing were categorized as either damaged or non-damaged based on their values. Lower DN_out_ values represent the non-damaged areas whereas higher values represent damaged areas.

### 3.4. Accuracy Assessment

**Figure 2.**
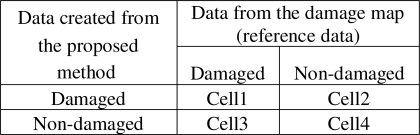
Accuracy assessment of each block.

The inner city of Adapazari was divided into ninety nine blocks of 300 m x 300 m size and these blocks were used for the detailed accuracy assessment procedure. The results obtained after the level slicing of the difference image (calculated from inverse Fourier transformed images) illustrates the damaged and non-damaged areas. These areas were compared with the 1/5,000 scale damage map for each block on a parcel basis to investigate the applicability of this method to automatically identify earthquake-induced damage. The number of parcels in each block was evaluated individually and error matrix for each block was created as shown in Figure 2.

Each block has four values corresponding to parcels identified as damaged both in the damage map and with the proposed method (cell 1), parcels identified as non-damaged both in the damage map and with the proposed method (cell 4), parcels identified as damaged in the damage map but non-damaged with the proposed method (cell 3) and parcels identified as non-damaged in the damage map but damaged with the proposed method (cell 2). Overall accuracy of each block was calculated by summing diagonal elements and dividing them to total number of parcels within that block. The equation of the overall accuracy for a block based on the values described in Figure 2 is as follows:

(5) $${\mathrm{Overall}\;\mathrm{accuracy}}_{\mathrm{BLOCK}(n)}=\frac{{\mathrm{CELL}1}_{\mathrm{BLOCK}(n)}+{\mathrm{CELL}4}_{\mathrm{BLOCK}(n)}}{{\mathrm{CELL}1}_{\mathrm{BLOCK}(n)}+{\mathrm{CELL}2}_{\mathrm{BLOCK}(n)}+{\mathrm{CELL}3}_{\mathrm{BLOCK}(n)}+{\mathrm{CELL}4}_{\mathrm{BLOCK}(n)}}$$

**Figure 3.**
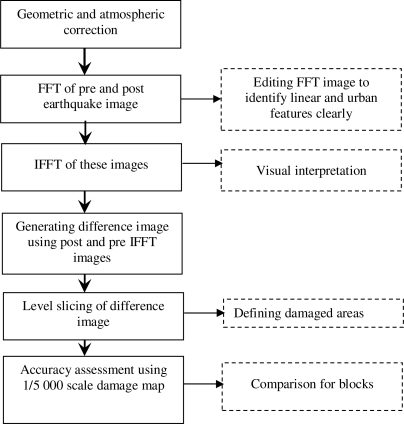
Procedures conducted in the methodology.

After calculating the overall accuracy of each block, total accuracy of the proposed method was calculated by rationing the sum of diagonals of all blocks to total number of parcels of all blocks. The performance of the proposed method was evaluated considering the total accuracy. Figure 3 shows the steps conducted during the study and this figure is also a summary of the methodology section.

## 4. Results

**Figure 4.**
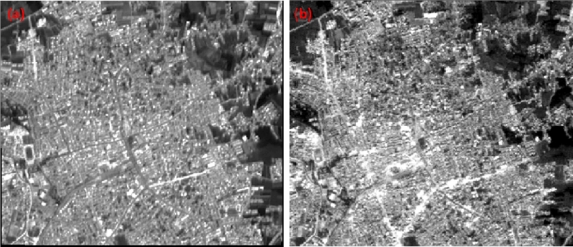

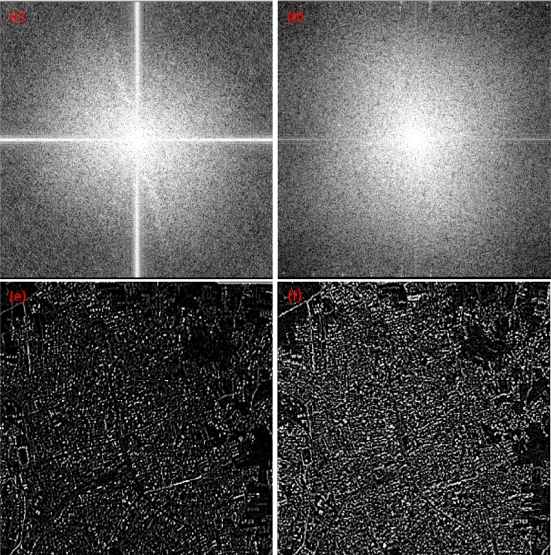
(a) Original pre-earthquake data, (b) original post-earthquake data, (c) FFT pre-earthquake image, (d) FFT post-earthquake image, (c) IFFT pre-earthquake image, (d) IFFT post-earthquake image.

The original pre- and post-earthquake images are shown in Figures 4 a and b and the FFT images generated from these data are illustrated in Figures 4 c and d. As a result of collapsed buildings and roads, spatial structure and texture of the post-earthquake image had changed. This caused differences in spatial frequency which can be determined via Fourier Transform. The differences in spatial frequency for pre- and post-earthquake data can be identified from Figures 4 c and d.

The low frequencies are plotted near the origin (center) while the higher frequencies are plotted further out. Generally, the majority of the information in an image is in the low frequencies indicated by the bright areas at the center of the Figures 4 c and d.

High pass filter was applied to satellite images in frequency domain to delineate the border of linear objects like roads and buildings precisely. Filters were applied to the low frequencies which are around the center for both pre- and post-earthquake data. After the filtering, IFFT was applied and edited Fourier images were converted back into the spatial domain.

**Figure 5.**
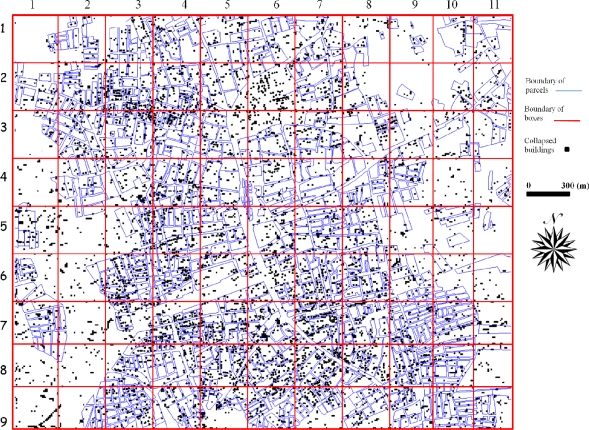
Blocks overlaid on the difference image (black: damaged areas, white: non-damaged areas)

After the visual interpretation of IFFT images, a difference image was generated by subtracting post-IFFT-image and pre-IFFT-image. Level slicing method with 10 slices was conducted to identify the damaged and non-damaged areas. Different numbers of levels were tried to find out the optimum number of slices and the analyses shows that having a slice number higher than 10 did not contribute significant information since only a few number of pixels was assigned to a slice. The histogram of the difference image was investigated to see the general distribution of data and to determine a threshold value for damaged and non-damaged regions. Further analyses were conducted with different threshold values to find out the most appropriate value for the study to identify the changes. Standard deviation (σ) and mean (µ) values obtained from the difference image were used for the analysis. 3σ, 2.5σ, 2σ, 1.8σ and 1.6σ, 1.5σ and1.4σ were tried and 1.4σ was found as the best threshold value to determine changes. Using this threshold value, slices including data between µ-1.4σ and µ+1.4σ were assigned as non-damaged areas whereas slices outside this range were assigned as damaged areas. Figure 5 shows the result of level sliced-difference image and blocks overlaid on this image with parcel boundaries.

**Figure 6.**
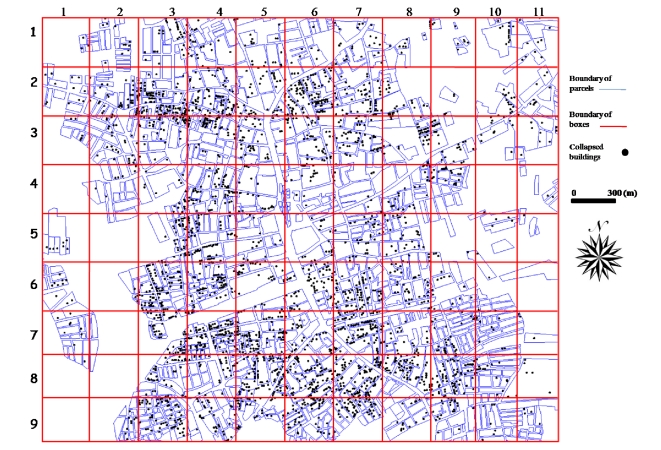
Blocks overlaid on the damage map.

Damaged areas obtained from remote sensing methods were compared with the 1/5,000 scale damage map to quantify the accuracy of the proposed method. Figure 6 shows the damage map and blocks where the detailed accuracy assessment was conducted and error matrixes created.

Comparisons were conducted for each block in parcel base. Number of damaged or non-damaged parcels within a block were calculated from difference image (Figure 5) and damage map (Figure 6). Table 2 includes each block with box number (BOX NO), box number are called based on their row and column location. For example, BOX NO 1–2 is corresponded to the box at row 1 and column 2 in Figure 5 and 6. Each block has four values corresponding to status of parcels as explained in Section 3.4. N/A is corresponded to not available meaning that there is either no parcel or damage data in those regions. Based on Table 2, the corresponding values of each cell for BOX 1–2 will be as following:

Cell 1: parcels identified as damaged both in damage map and with the proposed method, this value is 3 for block 1–2.
Cell 2: parcels identified as non-damaged in damage map but damaged with the proposed method, 4 for block 1–2.
Cell 3: parcels identified as damaged in damage map but non-damaged with the proposed method, 1 for block 1–2.
Cell 4: parcels identified as non-damaged both in damage map and with the proposed method, 6 for block 1–2.

In most cases parcels identified as damaged in the damage map but non-damaged with the proposed method (cell 3) occurred because there were only one or two collapsed buildings within a parcel which could not be identified using SPOT images. Block 1–2, 1–3, 1–10, 4–5 are examples of this situation. On the other hand, most of the parcels having more than three collapsed buildings were easily identified using the proposed method. Higher resolution satellite images should be used to identify collapsed buildings individually.

**Figure 7.**
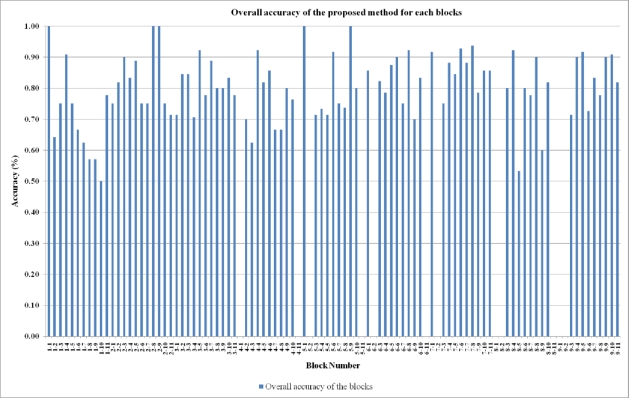
Overall accuracy the proposed method for each block.

The overall accuracy of each block ranges from 50 % to 100 % (Figure 7). Most of the blocks have accuracy value higher than 75 %. The minimum accuracy was obtained for Block 1–10, because there are three damaged parcels and two of these parcels include only one collapsed building which is hard to identify with current spatial resolution. Most of the parcels which have plenty of collapsed buildings were easily identified with 75 % or higher accuracy.

Total accuracy of the proposed method was calculated by rationing the sum of diagonals of all blocks to total number of parcels. The total accuracy obtained from this ratio is 80.19 %. The results illustrated that the proposed method can be successfully used to identify earthquake-induced damage areas automatically. Considering the spatial resolution of the satellite image used in this study, this accuracy value is reasonable. The results derived from this research can provide important information to many decision-makers and local authorities to determine location and magnitude of destructions and conduct emergency operations. However, depending on the end-user needs, if higher accuracy value is desired, high resolution satellite image should be used.

## 5. Conclusions

Remotely sensed data are crucial for disaster management, and rapid and reliable information extraction from these data is an important source for decision-making. Quick identification of heavily damaged areas in a disaster provides key information on potential damage and losses to buildings, transportation systems, industrial facilities and critical emergency facilities. These data can be used by urban planners and emergency managers to manage vulnerabilities of a region and develop risk mitigation plans.

This research proposed a new approach which is the integration of FFT and level slicing to accurately identify the location of the damaged areas caused by an earthquake. A difference image obtained from IFFT-pre- and post-earthquake data can provide significant information about the location and degree of earthquake induced damage areas. Most of the parcels which include more than three collapsed buildings were easily determined using the proposed method. The total accuracy of the method is calculated for pre-defined blocks in parcel basis and found to be 80.19 %. Higher resolution satellite images should be used to identify collapsed buildings individually. However, in some cases high resolution data of the region is not available and available satellite images of the region obtained before and after the disaster have to be used. Therefore, it is important to process the available data set rapidly and accurately and this research proposed an approach to fulfill this aim.

With the use of the proposed method, emergency managers, risk managers, and public policy/decision makers can understand the impact of earthquakes, identify the heavily damage areas to direct the emergency-response teams and incorporate the results into preparedness programs and early warning systems.

## Figures and Tables

**Figure 1.**
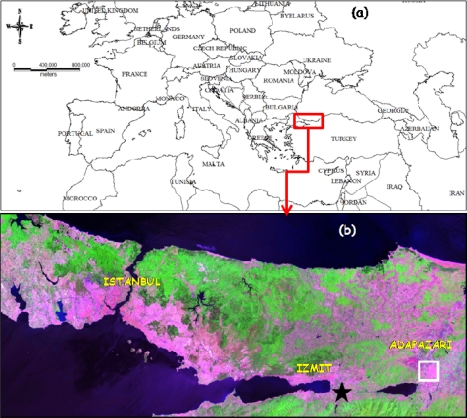
Location of the study area, (a) Turkey and surrounding countries, (b) Marmara Region, star shows the epicenter of the earthquake.

**Table 1.** Historical earthquakes in Turkey (USGS, 2008).

**Date**	**Location**	**Magnitude (Mw)**	**Fatalities**
17 August 1668	Anatolia	8.0	8,000
9 August 1912	Murefte (Ottomon Empire)	7.8	2,800
3 October 1914	Burdur (Ottoman Empire)	7.0	4,000
26 December 1939	Erzincan	7.8	32,700
26 November 1943	Ladik, Samsun	7.6	4,000
18 Mart 1953	Gonen	7.3	1,073
19 August 1966	Varto, Mus	6.8	2,529
28 March 1970	Gediz	6.9	1,086
30 October 1983	Erzurum	6.9	1,342
17 August 1999	Izmit	7.2	11,718

**Table 2.** Accuracy assessment of the proposed method using error matrixes.

**BOX NO**	**PARCELS**		**BOX NO**	**PARCELS**		**BOX NO**	**PARCELS**		**BOX NO**	**PARCELS**		**BOX NO**	**PARCELS**	
1–1	2	0	3-1	2	2	5-1	5	0	7-1	6	0	9-1	N/A	N/A
	0	1		0	3		0	1		1	5		N/A	N/A
1–2	3	4	3-2	8	1	5-2	N/A	N/A	7-2	N/A	N/A	9-2	N/A	N/A
	1	6		1	3		N/A	N/A		N/A	N/A		N/A	N/A
1–3	4	2	3–3	10	1	5-3	4	1	7-3	8	1	9-3	3	0
	1	5		1	1		1	1		2	1		2	5
1–4	7	1	3–4	6	1	5-4	8	1	7-4	13	1	9-4	6	0
	0	3		4	6		3	3		1	2		1	3
1–5	4	1	3–5	10	1	5–5	6	2	7-5	8	1	9-5	9	0
	1	2		0	2		2	4		1	3		1	2
1–6	5	3	3–6	5	1	5–6	3	0	7-6	7	0	9-6	4	1
	0	1		1	2		1	8		1	6		2	4
1–7	3	1	3–7	7	0	5–7	5	2	7–7	12	1	9-7	7	0
	2	2		1	1		1	4		1	3		2	3
1–8	2	1	3–8	5	1	5–8	9	4	7–8	8	0	9-8	4	0
	2	2		1	3		1	5		1	7		2	3
1–9	2	0	3–9	6	1	5–9	1	0	7–9	6	1	9-9	5	0
	3	2		1	2		0	4		2	5		1	4
1–10	1	2	3–10	4	0	5–10	3	1	7–10	4	1	9–10	4	0
	1	2		1	1		0	1		1	8		1	6
1–11	4	0	3–11	5	1	5–11	N/A	N/A	7–11	3	1	9–11	3	1
	2	3		1	2		N/A	N/A		0	3		1	6
2-1	6	1	4-1	N/A	N/A	6-1	3	1	8-1	N/A	N/A			
	1	0		N/A	N/A		0	3		N/A	N/A			
2–2	6	0	4-2	3	2	6-2	N/A	N/A	8-2	N/A	N/A			
	2	3		1	4		N/A	N/A		N/A	N/A			
2–3	6	0	4-3	4	2	6-3	10	0	8-3	5	1			
	1	3		1	1		3	4		1	3			
2–4	8	0	4–4	9	0	6-4	10	0	8-4	8	0			
	2	2		1	3		3	1		1	4			
2–5	6	0	4–5	7	2	6-5	6	1	8-5	5	0			
	1	2		0	2		0	1		7	3			
2–6	6	1	4–6	4	1	6–6	8	1	8-6	6	0			
	2	3		1	8		0	1		2	2			
2–7	4	0	4–7	2	1	6–7	10	1	8-7	5	0			
	2	2		2	4		3	2		2	2			
2–8	3	0	4–8	1	1	6–8	8	1	8–8	7	0			
	0	4		3	5		0	4		1	2			
2–9	2	0	4–9	5	0	6–9	5	1	8–9	4	1			
	0	0		3	7		2	2		3	2			
2–10	2	1	4–10	4	0	6–10	2	1	8–10	6	0			
	0	1		4	9		0	3		2	3			
2–11	4	0	4–11	N/A	N/A	6–11	N/A	N/A	8–11	N/A	N/A			
	2	1		N/A	N/A		N/A	N/A		N/A	N/A			
